# The Relationship Between Perceived Family Climate and Glycemic Control in Type 1 Diabetes Mellitus Adolescent Patients

**DOI:** 10.4274/jcrpe.3825

**Published:** 2017-09-01

**Authors:** Şafak Eray, Halit Necmi Uçar, Fatma Çetinkaya, Erdal Eren, Pınar Vural

**Affiliations:** 1 Van Training and Research Hospital, Clinic of Child and Adolescent Psychiatry, Van, Turkey; 2 Taksim Training and Research Hospital, Clinic of Child Health and Diseases, İstanbul, Turkey; 3 Uludağ University Faculty of Medicine, Department of Pediatric Endocrinology, Bursa, Turkey; 4 Uludağ University Faculty of Medicine, Department of Child and Adolescent Psychiatry, Bursa, Turkey

**Keywords:** Type 1diabetes mellitus, adolescents, perceived expressed emotion, glycemic control

## Abstract

**Objective:**

Type 1 diabetes mellitus (T1DM) is a chronic disease which ranks third in children under age 16 years. Expressed emotion (EE) is a term that indicates a specific family climate including lack of emotional support (LES), irritability, and emotional over-involvement. It is known that the family environment is highly important for glycemic control in diabetic adolescents. In this study, the relationship between perceived EE and glycemic control in adolescents diagnosed with T1DM not accompanied by psychopathology were investigated.

**Methods:**

The study included 49 adolescents with T1DM and 50 adolescents as a control group. Adolescents with psychopathology and intellectual disability were excluded from the study. Perceived EE was measured by the Shortened Level of Expressed Emotion Scale (SLEES) and blood sugar regulation was assessed by HbA1c levels.

**Results:**

The adolescents with T1DM showed a significant difference in perceived EE (p=0.020) and LES (p=0.014) when compared with the control group. When diabetic adolescents were compared among themselves, the diabetic adolescents with poor glycemic control perceived greater EE (p=0.033) and less emotional support (p=0.049). In regression analyses, the predictive power of mother’s educational level, the employment status of mothers and the subscale “LES” of SLEES combined to explain HbA1c level was determined to be 37.8%.

**Conclusion:**

The strong relationship between perceived EE and glycemic control showed us that perceived EE can hinder treatment compliance without causing psychopathology. For this reason, it is recommended that not only patients with psychopathology, but all diabetic adolescents receive psychosocial support and family interventions.

**What is already known on this topic?**

Expressed emotion has been linked to poor glycemic control in the literature. Although some studies have reported a positive or negative correlation between emotional over-involvement and glycemic control, there are also studies in which no correlation was found.

**What this study adds?**

In this study, the relationship between perceived expressed emotion and glycemic control in adolescents diagnosed with type 1 diabetes mellitus not accompanied by psychopathology will be investigated. To the best of our knowledge, no study concerning expressed emotion and glycemic control has been carried out in Turkey to date. We aimed to emphasize the role of family climate in the treatment of diabetic patients.

## INTRODUCTION

Adolescence is a transitional period from childhood to adulthood during which the individual has unique physical and psychological needs. Chronic illnesses such as diabetes mellitus hinder the physical and mental development of adolescents (1). Type 1 diabetes mellitus (T1DM) is a chronic disease which ranks third in frequency after asthma and cerebral palsy in children under 16 years of age (2). Chronic illnesses such as T1DM, which necessitate lifestyle changes, increase the likelihood of psychiatric disorders, including depression, in adolescents (3). This situation affects the compliance of patients with T1DM as well as the regulation of their blood sugar. It has been reported that family support is one of the most important indicators of compliance and treatment success (4,5,6,7,8).

Expressed emotion (EE) is an empirical concept that is accepted as a barometer of the emotional climate at home. This concept was formulated because of the strong relationship between environmental changes in the family system and the mental health of family members (9). EE is a measure of environmental stress at home, which is estimated by communication styles including the amount of criticism made by family members about the patients, the presence or absence of hostile attitudes, the level of intrusiveness, and emotional over-involvement (EOI) (10). EE is not only a predictor of psychological diseases but also may predict the state of physical disease (10). When the effects of family attitudes on glycemic control are taken into account, the relationship between T1DM and EE has captured the attention of the researchers (11,12,13,14,15). However, when the literature is examined, contradictory results are seen (12,13,14,15). In general, EE has been linked to poor glycemic control (11). Although some studies have reported a positive or negative correlation between EOI and glycemic control, there are also studies in which no correlation was found (12,13,14,15).

When these studies are examined, it is observed that the assessments were oriented toward the parents rather than the adolescents. Non-reliance on self-reporting by the adolescents and the failure to use assessments developed especially for adolescents can be viewed as a flaw of these studies. When the family climate is considered a reciprocal concept of emotional tone, the question of whether it is an individual characteristic of reciprocal interaction arises. How EE is perceived becomes important (16).

To the best of our knowledge, no studies concerning this subject have been conducted in Turkey to date. In this study, the relationship between perceived EE and glycemic control in adolescents diagnosed with T1DM not accompanied by psychopathology will be investigated. We aimed to emphasize the role of family climate in the treatment of diabetes.

## METHODS

The study was carried out with 99 adolescents-49 T1DM patients and 50 healthy control subjects. The patient group consisted of T1DM cases who had been diagnosed in the Uludağ University Pediatric Endocrinology Department at least one year prior to the study. These adolescents were living with both their father and mother and had no apparent disease or condition, such as the use of exogenous steroids, to disrupt their blood sugar regulation. Psychopathology and mental retardation were accepted as exclusion criteria. All participants in the study and their parents gave informed consent after being informed of the methods and objectives of this study. The necessary legal permission and approval were obtained from the Uludağ University Faculty of Medicine Ethics Committee before proceeding to the data collection stage.

The participants were initially requested to fill out the sociodemographic form prepared by the researchers and then were evaluated using the Affective Disorders and Schizophrenia Schedule for School Age Children Present and Lifetime Version (K-SADS-PL), a form for detection of psychopathology used by child and adolescent psychiatrists. Cases diagnosed to have a mental disorder such as depression, an anxiety disorder, or an attention deficit hyperactivity disorder were excluded. The participants were evaluated using the WISC-R test, and those whose scores were less than 85 were also excluded. The Shortened Level of Expressed Emotion Scale (SLEES) was filled out by the adolescents under supervision of the researchers. One additional participant was also excluded from the study because of unreliable answers. Blood sugar regulation over the previous three months was assessed by glycosylated hemoglobin (HbA1c). All patients were on multiple daily injections of insulin.

The control group of children was matched with the patient group with respect to age, gender, parental employment status, education, and family structure. The control group consisted of 50 adolescents with no psychopathology or mental retardation and was selected from among adolescents who had presented with physical complaints to the pediatric department and who volunteered to participate to the study.

Glycemic control in DM was assessed in accordance with standards suggested by the International Society for Pediatric and Adolescent Diabetes (ISPAD) in 2007, with optimal control defined as HbA1c <7.5%, suboptimal control as 7.5-9%, and poor control as >9% (17). The diabetic adolescents were divided into two groups based on their HbA1c levels, either above or below 9%, and each group was compared with the other group and the control group.

### Information Collection Form

The Information Collection Form was created by the researchers to collect information on the socio-demographic characteristics of the participants including age, gender, education level of the parents, employment status of the parents, number of siblings, birth order, economic status, as well as physical and mental health of family members. Socioeconomic status (SES) was determined based on the official starvation and poverty limits of 2015.

### Kiddie Schedule for Affective Disorders and Schizophrenia-Present and Lifetime Version (K-SADS-PL)

K-SADS-PL is a semi-structured diagnostic interview that was created to assess psychopathology in children and adolescents according to DSM-III and DSM-IV diagnostic criteria. It was developed by Kaufman et al (18) in 1997 and subsequently translated into Turkish. A validity and reliability study of the schedule for Turkish children was conducted by Gökler et al (19) in 2004. This tool serves to assess psychopathology in children and adolescents based on the data obtained by interviewing the child and his/her parents.

### Shortened Level of Expressed Emotion Scale in Adolescents

The scale developed by Nelis et al (20) was translated into Turkish by Vural et al (21) in 2013. SLEES consists of 33 items measuring the EE of the person perceived to be most important individual in the participant’s life over the previous 3 months. The three subscales of the SLEES include lack of emotional support (LES), irritability, and intrusiveness. Higher scores indicate higher levels of EE.

### Data Analysis

Data were evaluated using IBM Statistical Package for the Social Sciences Statistics 22 statistical software package program. The mean and the standard deviation values with minimum and maximum levels were used for the statistical expression of the groups. While the comparison of the continuous variables between the groups was performed using the student’s t-test for normally distributed variables, comparison of the abnormally distributed variables and non-parametric parameters was performed using the Mann-Whitney U test. For comparison of the categorical variables, a chi-square test was utilized. We used multiple linear regressions to examine HBA1C levels as predictors of the relationship between sociodemographic variables and EE scores. A p-value of <0.05 was considered statistically significant.

## RESULTS

The diabetic group and the control group were similar in age (14.46±1.24 years and 14.00±1.39 years, respectively) (t=1.763, p=0.081). Fifty-one percent (n=25) of the diabetic group and 60.0% (n=30) of the control group were girls.

To compare the income level, the poverty line defined as a monthly income under 3300 Turkish lira per month by the Turkish Statistical Institute was used, and the groups were further divided into high and low socio-economic status groups. In terms of family income, the difference between the patient and control groups was not statistically significant (χ²=2.541, p=0.111). Regarding the parents’ cohabitation, all participants were living with both parents. To compare educational status, the parents were divided into two groups, as those who completed high school and those who did not. No significant difference was found between the two groups (χ²=0.990, p=0.320) (χ²=0.093, p=0.761). Similarly, the employment status of the parents did not show a statistically significant difference (χ²=0.542, p=0.461) (χ²=2.926, p=0.087) (Table 1).

The SLEES scores of the patient and control groups are shown in Table 2. There was a significant difference in the total perceived EE scores between the groups and in the subscale of LES scores. There were no differences between the two groups in the subscales of irritability and intrusiveness.

Mean HbA1c of diabetic adolescents was 10.36±2.62%. The median value was 10.00, the minimum was 6.50, and the maximum value was 17.10. There was no statistically significant differences in HbA1c values of girls [standard deviation (SD)=10.57±2.39] and boys (SD=10.15±2.89) (t=0.547, p=0.587). In addition, there was no relationship between age and HbA1c values (p=0.368).

The diabetic patients were divided into two groups, those with HbA1c levels at or below 9% (group 1) and those with levels above 9% (group 2), to assess the relationship between perceived EE and glycemic control. The mean age of the two groups was similar (14.21±0.97 and 14.63±1.37 years, respectively). The female to male ratio in the two groups was 63.2% and 40.0%, respectively (p=0.114).

Means of the SLEES by groups were: X=54.8, SD=10.4 for group 1 and X=64.4, SD=19.6 for group 2. An independent sample t-test was conducted to evaluate the differences between the two groups. There were significant differences in the perceived EE scores of the groups (t=-2.199) (p=0.033). There was also a significant difference in the subscale of LES scores between the groups (t=-2.018) (p=0.049). There were no differences in the subscales of irritability and intrusiveness between the groups (Table 3).

To evaluate the variables that influence HbA1c levels of the diabetic adolescents, regression analysis was performed. Starting with 10 different variables, we proceeded by eliminating the least significant variables (Table 4). All models were found to be significant, but some variables were not. The significant variables were educational level of mothers, employment status of mothers, and subscale LES of SLEES. It was observed that maternal educational level was negatively associated with HbA1c. The employment status of mothers was positively associated with HbA1c. An increase in HbA1c was found when the LES subscale scores of SLEES increased. In conclusion, the combined predictive power of these three variables to explain HbA1c levels was determined to be 37.8%.

## DISCUSSION

In our study, adolescents with diabetes mellitus showed significantly higher perceived EE and perceived LES when compared with the control group. When diabetic adolescents were compared among themselves, the diabetic adolescents with poor glycemic control perceived greater EE and LES from their families.

Factors that may affect family climate, such as integrity of the family structure, a history of psychiatric disease, or newly diagnosed diabetes which may be associated with poor glycemic control, were accepted as exclusion criteria. During psychiatric evaluations, psychopathology, as expected, was observed to be more frequent in the diabetic group than in the control group. Adolescents with psychopathology were excluded from the study.

Mean HbA1c levels in our subjects were similar to those found in other studies and there were no significant differences with respect to gender and age, again consistent with the literature (22). When we examined the factors that influence glycemic control, it was found that low socio-economic level affected HbA1c levels negatively, as expected (23,24). Our study also showed that high maternal educational level affected glycemic control positively. However, paternal educational level did not have the same effect. Mothers with higher educational levels can deal better with diabetes and its treatment requirements, which include physical activity, proper diet, and insulin dosage. The role of mothers in family structure has been emphasized in previous studies (22,25). Father’s employment status was not determined to be an influencing factor. The results of our study were similar to those made in other countries. However, although the educational level of mothers was negatively associated with the Hba1c levels, the same was not true for maternal employment status. This situation can be explained by the lack of time that working mothers have to devote to regulating the lifestyle of their diabetic adolescents. However, this result, which may have been affected by sample size and socio-cultural characteristics, needs to be confirmed by further studies.

When diabetic adolescents and control groups were examined in terms of perceived EE, it was observed that diabetic adolescents perceived a higher EE than the control group. This result was mainly due to higher scores on the LES subscale, indicating that diabetic adolescents feel less emotional support. There are conflicting results in the literature concerning blood sugar regulation and EE. While Koenigsberg et al (12) stated that EE can predict glycemic control, Worrall-Davies et al (14) observed no relationship between the two. When the subscales of EE, EOI, and criticism were evaluated separately, there were also conflicting results regarding the relationship between glycemic control and EOI. While Stevenson et al (13) reported a positive association between glycemic control and EOI, Liakopoulou et al (15) found a negative correlation. Worrall-Davies et al (14) found no association between glycemic control and EOI. These conflicting results with regard to EOI in adolescents can be associated with the perception of EOI by adolescents. Excessive protection may be perceived negatively by adults, although children may perceive it in a positive way. No association between poor glycemic control and intrusiveness was found in our study. We also have observed no relationship between poor glycemic control and perceived irritability in our study.

In order to resolve the conflicting results regarding EOI, we used an assessment tool specially designed for adolescents, which had subscales including LES, irritability, and intrusiveness. We observed that LES and high EE were associated with poor glycemic control. There was no difference between the two groups in terms of irritability and intrusiveness.

The relationship between psychopathology and EE has been clearly demonstrated in several studies (26,27,28). It is also known that psychopathology rates are higher in patients with chronic diseases. Psychopathology affects glycemic control negatively, both because it is a source of chronic stress and because it tends to lower compliance rates. The aim of our study was to evaluate the effect of family climate on glycemic control by comparing diabetic adolescents without psychopathology to a control group.

The strengths of our study were the exclusion of psychopathology in both the control and diabetic groups based on clinical interviews and evaluating EE by using a self-report scale specially designed for adolescents. Limitations of our study were not evaluating for psychopathology in other family members by psychiatrists and the low number of the diabetic patients.

The strong relationship between perceived EE and glycemic control showed that it can hinder treatment compliance without causing psychopathology. For this reason, not only patients with psychopathology, but all diabetic adolescents require psychosocial support and family interventions. As psychiatric consultation may play a critical and positive role in the treatment of chronic diseases, we recommend that psychosocial interventions be part of any diabetes treatment. Although most diabetics do not have psychiatric disorders, psychiatric intervention is recommended to improve compliance by the diabetics themselves and their families. We suggest that our study be repeated with a larger sample size and longer follow-up with family intervention, as such studies are lacking in the literature.

## Figures and Tables

**Table 1:**
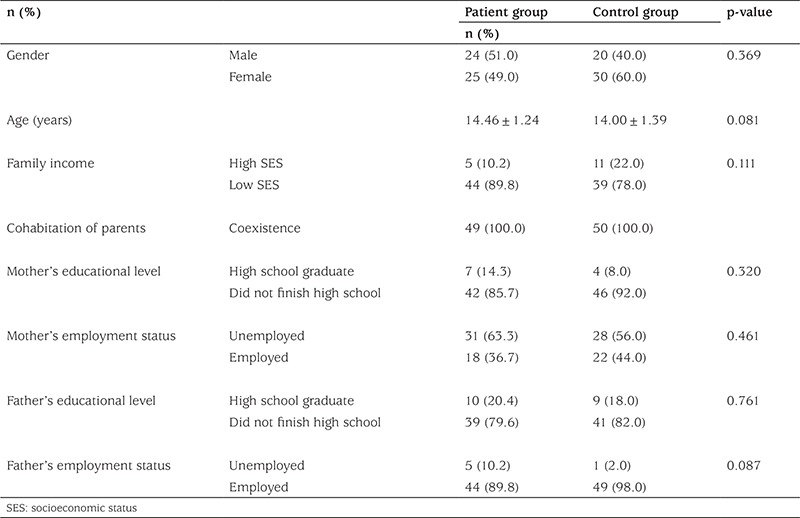
Sociodemographic variables of the participants

**Table 2:**
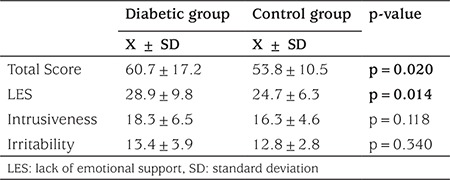
Expressed emotion scores in the diabetic group and control group

**Table 3:**
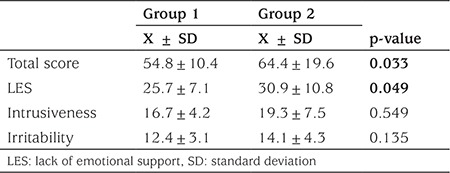
Relationship between perceived expressed emotion and glycemic control in the diabetic group

**Table 4:**
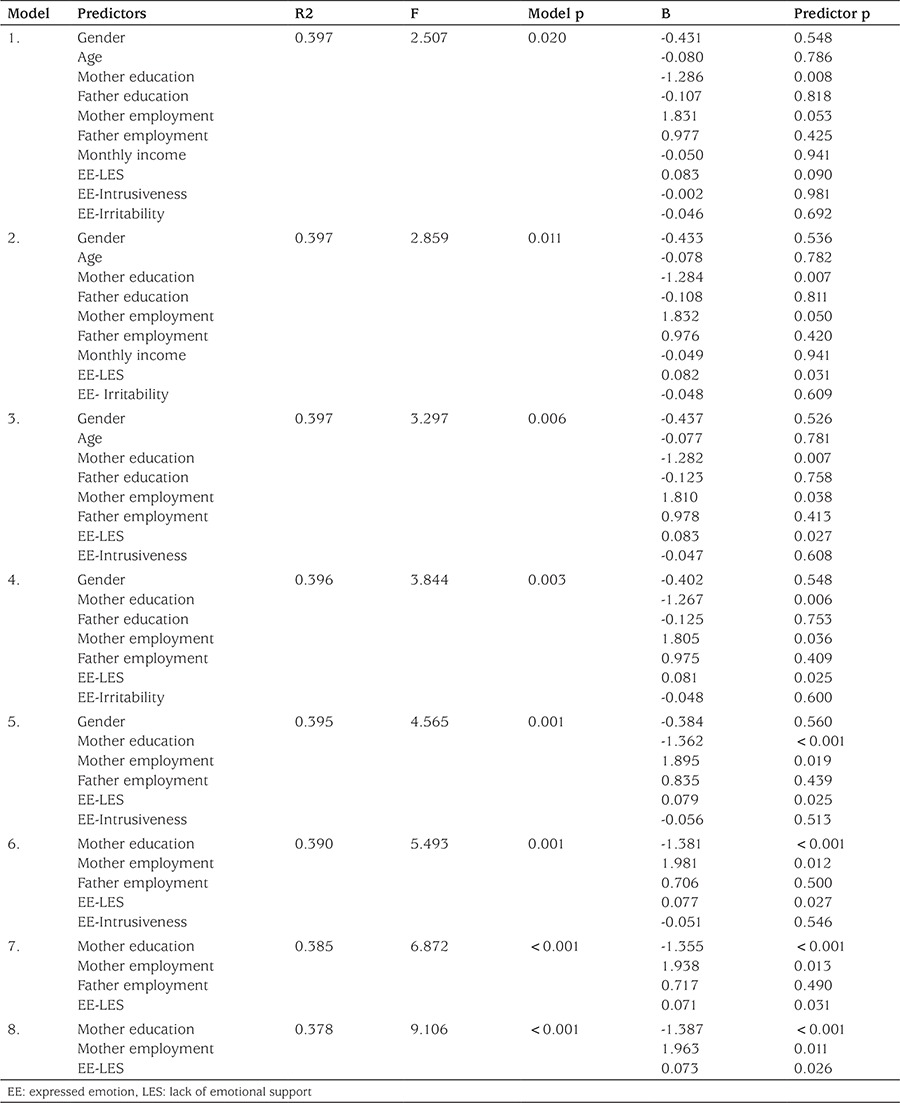
Regression analysis of the variables influencing HbA1c
